# Supplementary material for: Subtelomeric 5-enolpyruvylshikimate-3-phosphate synthase copy number variation confers glyphosate resistance in Eleusine indica
Source: Nat Commun. 2023 Aug 11;14:4865. doi: 10.1038/s41467-023-40407-6 (PMC10421919; doi:10.1038/s41467-023-40407-6)
Supplement: Supplementary file 1 — Supplementary Information [file 41467_2023_40407_MOESM1_ESM.pdf]

Subtelomeric *5-enolpyruvylshikimate-3-phosphate synthase* copy number variation confers glyphosate resistance in *Eleusine indica*

Zhang *et al*.

Supplementary Table 1. Assembly statistics.

	Susceptible assembly		Resistant assembly	
	**Number**	**Percentage**	**Number**	**Percentage**
Number of scaffolds	109		63	
Total size of scaffolds	522,557,097		560,916,264	
Longest scaffold	70,926,600		71,620,515	
Shortest scaffold	996		106,467	
Number of scaffolds > 1K nt	107	98.2%	63	100.0%
Number of scaffolds > 10K nt	100	91.7%	63	100.0%
Number of scaffolds > 100K nt	43	39.4%	63	100.0%
Number of scaffolds > 1M nt	10	9.2%	193	0.2%
Number of scaffolds > 10M nt	9	8.3%	12	19.0%
Mean scaffold size	4,794,102		8,903,433	
Median scaffold size	68,736		260,652	
N50 scaffold length	57,376,714		51,198,921	
L50 scaffold count	5		5	
scaffold %A		27.9%		28.0%
scaffold %C		22.1%		22.0%
scaffold %G		22.1%		22.1%
scaffold %T		27.9%		27.9%
scaffold %N		0.0%		0.0%
scaffold %non-ACGTN		0.0%		0.0%
Number of scaffold non-ACGTN nt	0		0	
Percentage of assembly in scaffolded contigs		58.7%		0.0%
Percentage of assembly in unscaffolded contigs		41.3%		100.0%
Average number of contigs per scaffold	2		1	
Number of contigs	171		63	
Number of contigs in scaffolds	88		0	
Number of contigs not in scaffolds	83		63	
Total size of contigs	522,552,240		560,916,264	
Longest contig	70,926,600		71,620,515	
Shortest contig	383		106,467	
Number of contigs > 1K nt	168	98.2%	63	100.0%
Number of contigs > 10K nt	158	92.4%	63	100.0%
Number of contigs > 100K nt	64	37.4%	63	100.0%
Number of contigs > 1M nt	15	8.8%	19	30.2%
Number of contigs > 10M nt	13	7.6%	12	19.0%
Mean contig size	3,055,861		8,903,433	
Median contig size	67,250		260,652	
N50 contig length	42,109,816		51,198,921	
L50 contig count	5		5	

contig %A		27.9%		28.0%
contig %C		22.1%		22.0%
contig %G		22.1%		22.1%
contig %T		27.9%		27.9%
contig %N		0.0%		0.0%
contig %non-ACGTN		0.0%		0.0%
Number of contig non-ACGTN nt	0		0	

Supplementary Table 2. Benchmarking Universal Single-Copy Orthologs (BUSCO) completeness and LTR Assembly Index (LAI) scores of glyphosate-susceptible and -resistant *Eleusine indica* using the embryophyta_odb10 BUSCO database.

**Phenotype**	**Data**	**BUSCO score**	**LAI score**
Glyphosate-susceptible	Falcon assembly	96.6%	
Glyphosate-susceptible	Hi-C mapping	98.0%	
Glyphosate-susceptible	PBjelly gap filling	98.1%	
Glyphosate-susceptible	Arrow error corrections	98.1%	
Glyphosate-susceptible	Final assembly	97.8%	18.77
Glyphosate-susceptible	Genome Annotation	92.1%	
Glyphosate-resistant	HiCanu assembly	97.8%	16.85
Glyphosate-resistant	Genome Annotation	92.2%	

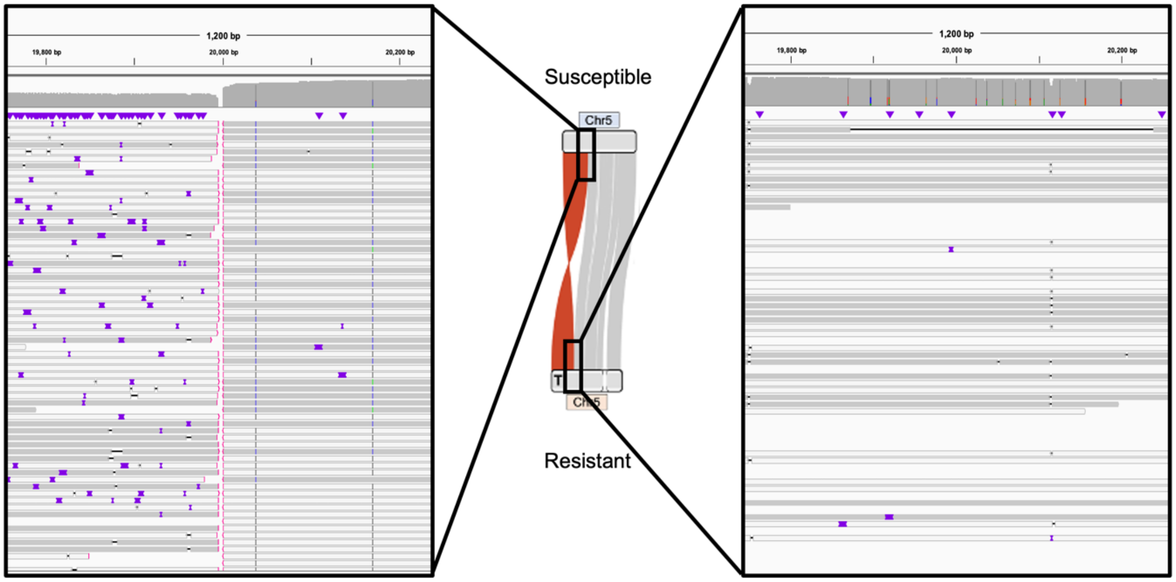

Resistant genome reads aligned to the susceptible genome Chr5 inversion point.

Resistant genome reads aligned to the resistant genome Chr5 Inversion point.

Supplementary Fig. 1. Confirmation of the inversion in chromosome five. This alignment shows a snapshot of 100,000 genomic PacBio reads from the resistant genome library that span the inversion junction on chromosome five. Reads do not span the inversion junction from the glyphosate-susceptible assembly, however they do span the inversion junction from the glyphosate-resistant assembly.

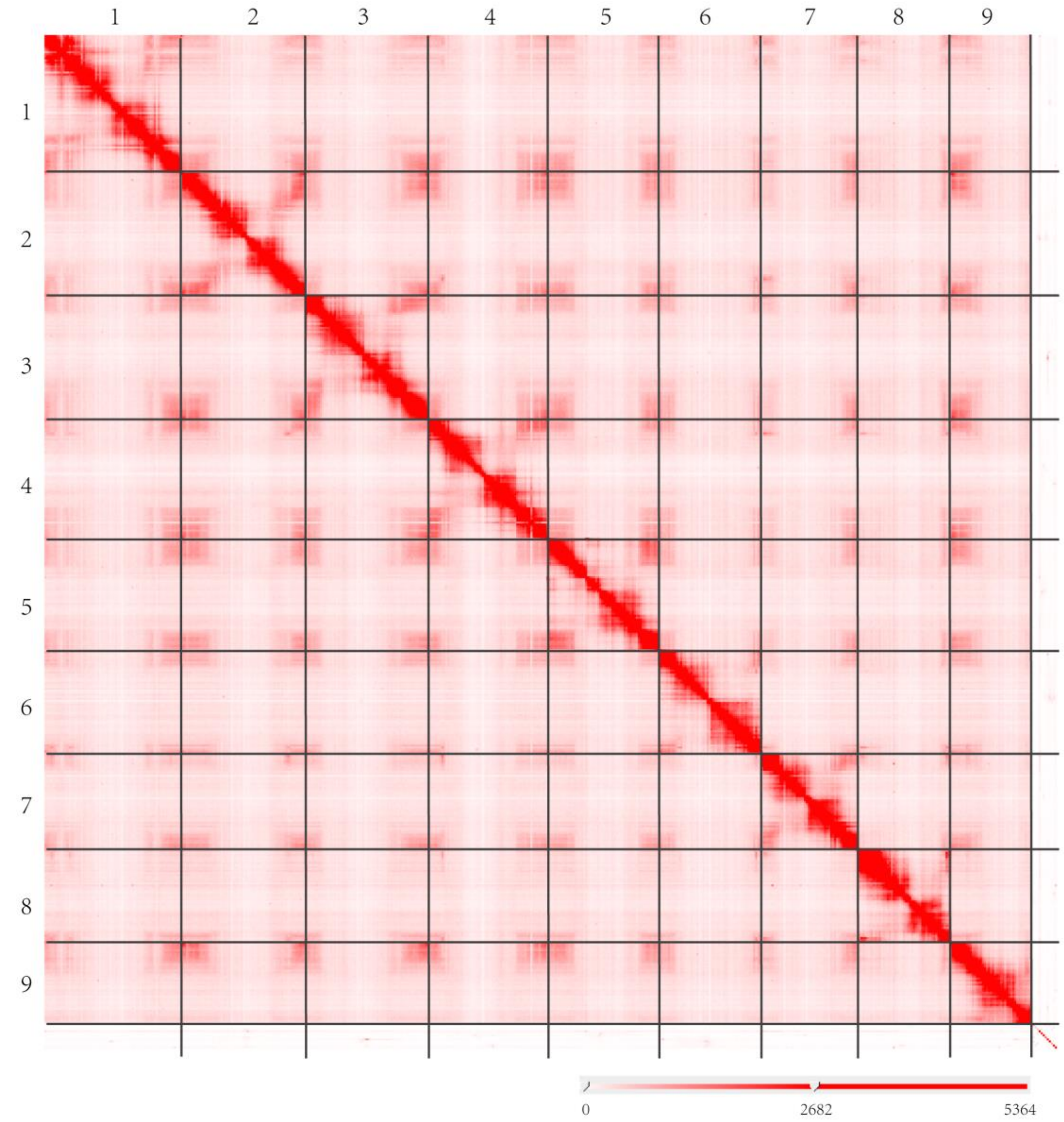

Supplementary Fig. 2. Hi-C contact map of the glyphosate-susceptible genome. Contact matrices were generated by aligning a Hi-C data to the glyphosate-susceptible assembly. Pixel intensity corresponds to the frequency of loci colocalization.

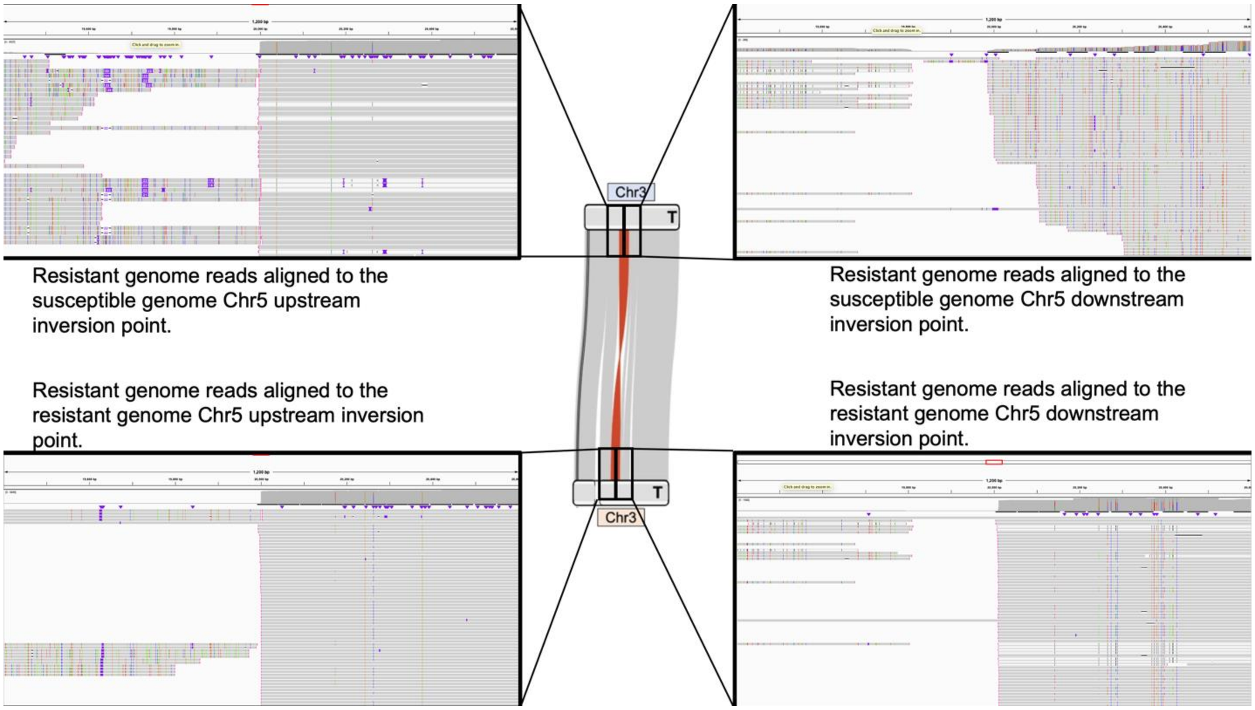

Supplementary Fig. 3. Confirming the inversion in chromosome three. This alignment shows a snapshot of 100,000 genomic PacBio reads from the resistant genome library that span the inversion junctions upstream and downstream chromosome three. Significant numbers of reads do not span any of the inversion junctions, upstream or downstream from either the glyphosate-resistant or glyphosate-susceptible genome assemblies.

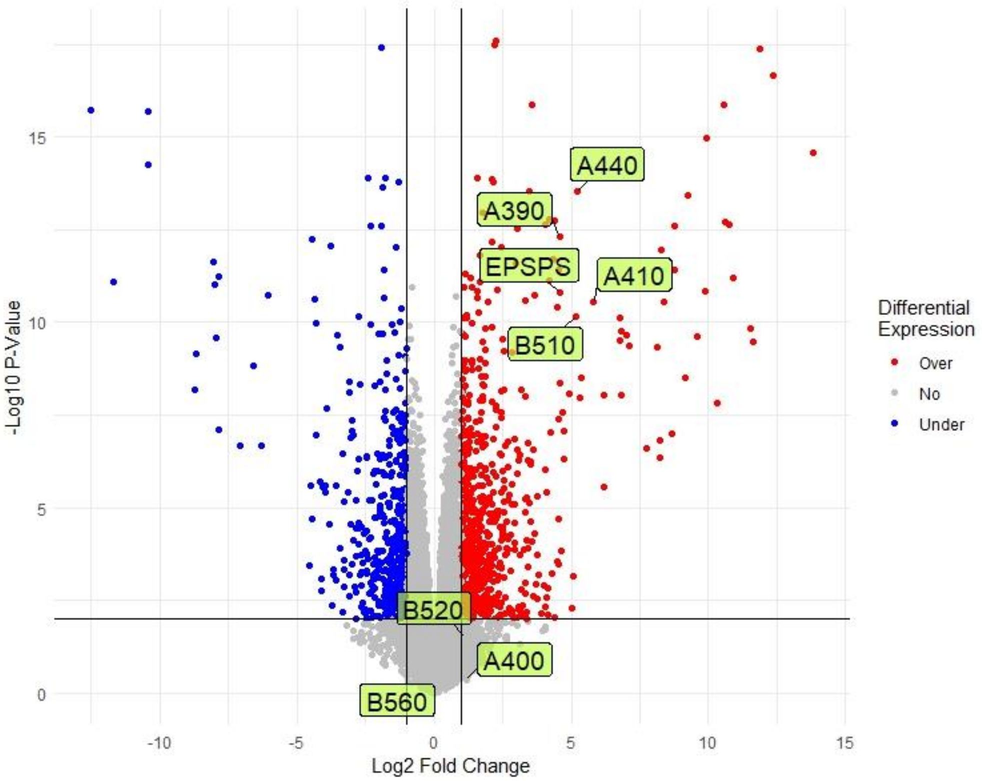

Supplementary Fig. 4. Differential expression of eight glyphosate-resistnt versus eight glyphosate-susceptible *Eleusine indica* individuals. The plot from RNA-Seq data shows over-expressed (red) and under-expressed (blue) genes in GR *E. indica* individuals with labels for all identified genes within the EPSPS cassette. Genes below a *p*-value of 0.01 or a fold change value below two were considered not differentially expressed (grey) between the two treatment groups.
