# Supplementary material for: Subtelomeric 5-enolpyruvylshikimate-3-phosphate synthase copy number variation confers glyphosate resistance in Eleusine indica
Source: Nat Commun. 2023 Aug 11;14:4865. doi: 10.1038/s41467-023-40407-6 (PMC10421919; doi:10.1038/s41467-023-40407-6)
Supplement: Supplementary file 3 — Reporting Summary [file 41467_2023_40407_MOESM3_ESM.pdf]

nature portfolio

Corresponding author(s): Eric Patterson, Qin Yu, Xingshan Tian

Last updated by author(s): Feb 27, 2023

Reporting Summary

Nature Portfolio wishes to improve the reproducibility of the work that we publish. This form provides structure for consistency and transparency in reporting. For further information on Nature Portfolio policies, see our Editorial Policies and the Editorial Policy Checklist.

Statistics

For all statistical analyses, confirm that the following items are present in the figure legend, table legend, main text, or Methods section.

n/a	Confirmed	
☐	☒	The exact sample size (*n*) for each experimental group/condition, given as a discrete number and unit of measurement
☒	☐	A statement on whether measurements were taken from distinct samples or whether the same sample was measured repeatedly
☒	☐	The statistical test(s) used AND whether they are one- or two-sided<br>*Only common tests should be described solely by name; describe more complex techniques in the Methods section.*
☒	☐	A description of all covariates tested
☒	☐	A description of any assumptions or corrections, such as tests of normality and adjustment for multiple comparisons
☒	☐	A full description of the statistical parameters including central tendency (e.g. means) or other basic estimates (e.g. regression coefficient) AND variation (e.g. standard deviation) or associated estimates of uncertainty (e.g. confidence intervals)
☒	☐	For null hypothesis testing, the test statistic (e.g. *F*, *t*, *r*) with confidence intervals, effect sizes, degrees of freedom and *P* value noted<br>*Give P values as exact values whenever suitable.*
☒	☐	For Bayesian analysis, information on the choice of priors and Markov chain Monte Carlo settings
☒	☐	For hierarchical and complex designs, identification of the appropriate level for tests and full reporting of outcomes
☒	☐	Estimates of effect sizes (e.g. Cohen's *d*, Pearson's *r*), indicating how they were calculated

Our web collection on statistics for biologists contains articles on many of the points above.

Software and code

Policy information about availability of computer code

Data collection	*Provide a description of all commercial, open source and custom code used to collect the data in this study, specifying the version used OR state that no software was used.*
Data analysis	Falcon (version 0.5)<br>Arrow (version 1.0)<br>Juicer (version 1.6)<br>PBjelly (version 1.0)<br>Pilon (version 1.24)<br>BLAST (version 2.9.0)<br>BUSCO (version 5.4.2)<br>Fastp (version 0.20)<br>HiCanu (version 2.1)<br>RepeatModeler (version 2.0.2)<br>RepeatMasker (version 4.1.2)<br>bedtools (version 2.30.0)<br>Minimap2 (version 2.24)<br>SAMtools (version 1.11)<br>cDNA Cupcake (version 28.0)<br>MAKER (version 3.01.04)<br>AGAT (version 0.8.0)

gffread (version 0.12.7)
MMseqs2 (version 4.1)
InterProScan 5 (version 5.47-82.0)
MultiLoc2 (version 1.0)
HiSat2 (version 2.1.0)
CNVnator (version 0.4.1)
edgeR (version 3.38.1); R (version 4.2.0)
YASS (version 1.16)
Circos (version 0.69-9)
RIdeogram (version 0.2.2); R (version 4.2.2)
ggplot2 (version 3.4.0); R (version 4.2.2)
RAXML-NG (version 1.1.0)
cowplot (version 1.1.1); R (version 4.2.2)

For manuscripts utilizing custom algorithms or software that are central to the research but not yet described in published literature, software must be made available to editors and reviewers. We strongly encourage code deposition in a community repository (e.g. GitHub). See the Nature Portfolio guidelines for submitting code & software for further information.

Data

Policy information about availability of data

All manuscripts must include a data availability statement. This statement should provide the following information, where applicable:

- Accession codes, unique identifiers, or web links for publicly available datasets
- A description of any restrictions on data availability
- For clinical datasets or third party data, please ensure that the statement adheres to our policy

The assembled genomes, associated GFF annotation files, and all functional annotation information are publicly available through the International Weed Genomics Consortium online database, 'Weedpedia' (https://weedpedia.weedgenomics.org/). Raw sequencing data has been submitted to the National Center for Biotechnology Information (NCBI) Sequence Read Archive (SRA); Genome resequencing data with the accession numbers SRR23364316-SRR23364331 and RNA-seq data with the accession numbers SRR23372273-SRR23372288.

Human research participants

Policy information about studies involving human research participants and Sex and Gender in Research.

Reporting on sex and gender	None.
Population characteristics	None.
Recruitment	None.
Ethics oversight	None. No human participants used in this research.

Note that full information on the approval of the study protocol must also be provided in the manuscript.

Field-specific reporting

Please select the one below that is the best fit for your research. If you are not sure, read the appropriate sections before making your selection.

[x] Life sciences [ ] Behavioural & social sciences [ ] Ecological, evolutionary & environmental sciences

For a reference copy of the document with all sections, see nature.com/documents/nr-reporting-summary-flat.pdf

Life sciences study design

All studies must disclose on these points even when the disclosure is negative.

Sample size	8 resistant and 8 susceptbale individuals from two populations
Data exclusions	*Describe any data exclusions. If no data were excluded from the analyses, state so OR if data were excluded, describe the exclusions and the rationale behind them, indicating whether exclusion criteria were pre-established.*
Replication	8 biological replicates per population
Randomization	This is a binary comparison between two distinct populations. Plants were grown in the same growth space.
Blinding	None.

Reporting for specific materials, systems and methods

We require information from authors about some types of materials, experimental systems and methods used in many studies. Here, indicate whether each material, system or method listed is relevant to your study. If you are not sure if a list item applies to your research, read the appropriate section before selecting a response.

Materials & experimental systems

n/a	Involved in the study
☒	☐ Antibodies
☒	☐ Eukaryotic cell lines
☒	☐ Palaeontology and archaeology
☐	☒ Animals and other organisms
☒	☐ Clinical data
☒	☐ Dual use research of concern

Methods

n/a	Involved in the study
☒	☐ ChIP-seq
☒	☐ Flow cytometry
☒	☐ MRI-based neuroimaging

Animals and other research organisms

Policy information about studies involving animals; ARRIVE guidelines recommended for reporting animal research, and Sex and Gender in Research

Laboratory animals	None.
Wild animals	None.
Reporting on sex	None.
Field-collected samples	One glyphosate-susceptible (GS) and one glyphosate-resistant (GR) population of E. indica that were previously characterized by Zhang et al., 2021 (DOI: 10.1002/ps.6527) were collected from Guangdong Province, China. These populations were purified and self-pollinated for increased homozygosity and consistency of GS or GR phenotypes. The GR population was confirmed to have EPSPS copy number variation by DNA quantitative PCR before purification.<br>Seeds of purified GS and GR biotypes were sown on wet filter paper in Petri dishes in a climate chamber at 28–30 °C, with 12h/12h light/dark period and 70% relative humidity. The two-leaf stage seedlings were transplanted into 28 × 54 cm trays (50 plants per tray) filled with potting soil and grown in a glasshouse. At the tillering stage, about ten individuals were randomly selected each from the GS and GR E. indica population and characterized. Three tillers of each plant were separated and repotted (one tiller per pot, 60 pots in total). One tiller of each plant was used for glyphosate resistance and susceptibility phenotyping, one for EPSPS CNV estimation and one for subsequent sequencing.<br>For glyphosate resistance and susceptibility phenotyping, one regrowth tiller (three days after tiller cloning) was treated with commercial glyphosate (41% glyphosate isopropylamine salt, 400 g ai ha-1 for GS and 1600 g ai ha-1 for GR), and GR (i.e., survivors) and GS (i.e., killed) phenotypes were determined three weeks after treatment. EPSPS CNV was again assessed in the GR population to ensure the CNV event was still present before genomic work began. Leaf material from untreated tillers of corresponding resistant and susceptible plants was used for genomic DNA isolation using the Plant Genomic DNA kit (Trans Gen Biotech Beijing Co., LTD). Quantitative PCR was performed using published primer pairs and methods where EPSPS copy number was compared to the single copy acetolactate synthase (ALS) gene as the internal reference. The untreated tiller of a confirmed GS plant was used for genomic sequencing performed at Shanghai OE Biotech Co., Ltd (Shanghai, China).
Ethics oversight	None was required. Only a weedy plant species was used in this research.

Note that full information on the approval of the study protocol must also be provided in the manuscript.
